# On Aerobic Exercise and Behavioral and Neural Plasticity

**DOI:** 10.3390/brainsci2040709

**Published:** 2012-11-29

**Authors:** Rodney A. Swain, Kiersten L. Berggren, Abigail L. Kerr, Ami Patel, Caitlin Peplinski, Angela M. Sikorski

**Affiliations:** 1 Department of Psychology, University of Wisconsin-Milwaukee, Milwaukee, WI 53211, USA; Email: kiersten@uwm.edu (K.L.B.); amipatel@uwm.edu (A.P.); peplin27@uwm.edu (C.P.); 2 Department of Psychology, Illinois Wesleyan University, Bloomington, IL 61702, USA; Email: akerr@iwu.edu; 3 Department of Psychology, Texas A & M University-Texarkana, Texarkana, TX 75503, USA; Email: angela.sikorski@tamut.edu

**Keywords:** experience-dependent plasticity, brain, angiogenesis, neurogenesis, depression, anxiety, learning and memory

## Abstract

Aerobic exercise promotes rapid and profound alterations in the brain. Depending upon the pattern and duration of exercise, these changes in the brain may extend beyond traditional motor areas to regions and structures normally linked to learning, cognition, and emotion. Exercise-induced alterations may include changes in blood flow, hormone and growth factor release, receptor expression, angiogenesis, apoptosis, neurogenesis, and synaptogenesis. Together, we believe that these changes underlie elevations of mood and prompt the heightened behavioral plasticity commonly observed following adoption of a chronic exercise regimen. In the following paper, we will explore both the psychological and psychobiological literatures relating to exercise effects on brain in both human and non-human animals and will attempt to link plastic changes in these neural structures to modifications in learned behavior and emotional expression. In addition, we will explore the therapeutic potential of exercise given recent reports that aerobic exercise may serve as a neuroprotectant and can also slow cognitive decline during normal and pathological aging.

## 1. The Effects of Exercise on Humans

The human brain has the potential for neuroplasticity. One such environmental contributor to the brain’s ability to change both structurally and functionally is exercise. Many physiological effects that underlie neuroplasticity also contribute to a number of cognitive processes, thus leading to the notion that exercise benefits not only the function of the body, but that of the brain as well. In humans, the majority of the research on this topic focuses on aging populations in an effort to identify strategies for promoting healthy cognitive aging and reduce age-related declines in learning and memory. However, exercise proves to be beneficial to cognitive performance throughout the lifespan. Here we will consider the impact of exercise across the lifespan, beginning with the long-term effects of exercise in children. We will also consider both the acute and chronic effects of exercise in young and aging adults as well as in situations of brain injury and mental health. 

When considering human studies of exercise and cognition, there are a few important things to remember. First, while it is well understood that exercise correlates with better cognitive function, there is a good deal of variability in the research findings, which is most likely attributable to methodological variations between studies, including the age range of participants, intensity, and length of exercise interventions, and previous activity levels of participants [1]. In addition, the nature of experimental and control groups sometimes differ across studies, with control groups consisting of non-exercisers, non-aerobic exercisers that engage in strength and conditioning training, and non-aerobic exercisers that engage in flexibility training. Similarly, many studies have high rates of participant attrition, which may result in variable or inconsistent experimental outcomes such that it is difficult to draw valid conclusions regarding the relationship between exercise and cognitive function. We will address these issues further as we proceed with our discussion.

### 1.1. Impact of Exercise on the Developing Brain

#### 1.1.1. Affect

Physical activity affects mental health and psychological well-being in children and adolescents. In both male and female adolescents there is a significant inverse relationship between physical activity and depression [2,3], and physical activity and anxiety [2]. Physical activity is also negatively correlated with self-esteem [2,4]. Exercise intervention appears to improve mood in depressed children and adolescents. A four-year longitudinal study found a significant negative relationship between physical activity and depression in adolescent girls who had been diagnosed with a mood disorder at baseline [5]. They also found that depressed female adolescents who jogged as a part of therapy exhibited significantly lower levels of depression when compared to those who did not exercise [6]. 

#### 1.1.2. Cognition

Aerobic exercise has also been linked to a number of changes in cognitive and neural development in children. Exercising and being physically fit are associated with better grades in school [7,8,9] whereas being overweight or obese, often part of a more sedentary lifestyle, are associated with poorer academic achievement [7,10]. In a meta-analysis of 44 studies on the effects of physical activity on cognition in children aged 4–18, Sibley and Etnier [11] found that there was a significant relationship between physical activity and improved performance on tests of perceptual skills, developmental level/academic readiness, intelligence quotient (IQ), academic achievement, mathematics, and verbal abilities. Though all age groups appeared to benefit from exercise, the strongest association between physical activity and cognitive function was found in children aged 4–7. These data point to the strong relationship between exercise and a variety of cognitive processes in children of all ages and highlight the specific importance of physical activity particularly in young children. 

On standardized neuropsychological measures, executive functions appear to be the most highly associated with exercise [12]. In one study, 38 children were divided into higher-fit and lower-fit groups based on their performance on a field test of aerobic fitness [13]. The children performed a Flanker task, which requires varying levels of executive control, while event-related brain potential (ERP) responses were measured. Findings indicated that higher-fit children had more accurate performance across the various conditions of the Flanker task, though there were no group differences in the reaction time. In addition, the ERP data indicated that the higher-fit children had a larger P3 amplitude across all conditions of the Flanker task compared to lower-fit children. The P3 amplitude is believed to index the brain activity necessary to maintain working memory and therefore is said to be proportional to the amount of attentional resources allocated to a given task [14]. Hillman *et al.* [13] also found that across all conditions of a flanker task, higher-fit children had a significantly greater P3 amplitude compared to lower-fit children. Furthermore, compared to lower-fit children, the negative amplitude of the wave related to error was smaller in higher-fit children, and these children also had increased positive error-related amplitude. These results suggest that during the initial learning of the task, higher-fit children are better able to attend to the relevant information, and thus experience less interference when the time comes to make the response. All of these factors lead higher-fit children’s performance to be superior to their less-fit peers. Hillman and colleagues have also reported that higher levels of physical fitness are associated with faster cognitive processing speeds and better performance on the Stroop and Flanker tasks [13,15,16]. 

While the previously mentioned research suggests a strong relationship between physical activity and cognitive performance, the limitation of these studies is the failure to utilize a true experimental design. However, studies of exercise and cognition that can be classified as truly experimental have found that exercise does have a significant effect on cognitive performance. Davis and colleagues showed that the neuroanatomical changes in the brain associated with enhanced performance on cognitive tasks can be visualized and localized. One hundred seventy-one overweight, sedentary 7- to 11-year-old children were randomly assigned to either an exercise program or a no exercise control group for approximately 13 weeks [17]. Children in the exercise group were further divided into high-dose (40 min/day) or low-dose (20 min/day) exercise groups. Functional magnetic resonance imaging (fMRI) was used to evaluate brain activation while the children completed tests of executive function. As in previous studies [18], results indicated that there was a significant dose-response related benefit of exercise on executive functions as well as mathematics achievement on the Woodcock-Johnson Tests of Achievement III. Further, fMRI results indicated that there was increased bilateral prefrontal cortex activity and reduced bilateral posterior activity in the children who exercised, indicating that exercise preferentially impacted areas of the brain necessary for higher cognitive functioning. 

Subcortical structures have also been demonstrated to be affected by physical activity, though these structures have been less researched in humans. Consistent with the animal literature, the human hippocampus appears to benefit from exercise. In one behavioral study, higher-fit and lower-fit children were asked to study faces and houses under relational and non-relational encoding conditions [19]. Results indicated that higher-fit children performed selectively better on the relational encoding condition. In addition, magnetic resonance imaging (MRI) has revealed larger hippocampal [20] and dorsal striatal [21] volumes in higher-fit children that were associated with better relational memory and Flanker task performance. Because relational memory is dependent on the prefrontal-hippocampal circuitry, these results provide behavioral support for the claim that exercise improves subcortical neural function associated with learning and memory output. 

Perhaps what is most significant about physical fitness in children is that there is evidence that aerobic exercise in childhood might increase the resiliency of the brain later in life, a phenomenon referred to as “cognitive reserve” (for review, see [22]). According to some researchers, physical fitness increases the brain’s resilience to neurological damage. Though speculative at this point, the cognitive reserve might result from enhanced development of the cerebral cortex during childhood, which might then provide long-lasting changes in brain structure and function later in life [23].

### 1.2. Impact of Exercise on the Adult Brain

#### 1.2.1. Affect

The therapeutic role of aerobic exercise in mental health has long been recognized [24,25,26]. In fact, evidence suggests that sedentary behavior may be associated with mental disorders such as depression and anxiety disorders. For instance, according to a survey of 1900 healthy individuals between the ages of 25–77 years, physical inactivity was found to be a risk factor for depressive symptoms [27]. Another study conducted using a community sample of 1536 individuals ages 15 and up found that the odds ratio for depression was significantly higher for the physically inactive participants compared to participants that exercised regularly [28]. In a sample of 19,288 individuals, lower levels of depression, anxiety, and neuroticism were observed in the individuals who exercised regularly [29]. Importantly, data from cross-sectional and prospective-longitudinal clinical and epidemiological studies indicates that there is a direct relationship between physical inactivity and symptoms of depression and anxiety [30]. 

##### 1.2.1.1. Depression

A number of studies have reported that exercise may reduce symptoms of depression in both nonclinical and clinically depressed populations. In a meta-analysis of 80 studies, exercise was associated with depression scores approximately half a standard deviation lower than control groups [31]. In another meta-analysis of 30 studies using only participants diagnosed with clinical depression, the length of the exercise intervention was reported to be a significant moderator for depressive symptoms [32]. That is, interventions that lasted nine weeks or more were associated with larger reductions in depressive symptoms. A meta-analysis of 14 randomized controlled trials comparing exercise intervention groups to non-treatment control groups reported that exercise was just as effective as cognitive therapy in amelioration of depressive symptoms [33].

In fact, multiple studies have reported that exercise may be used as a first-line of treatment for mild to moderate symptoms of depression. In one randomized controlled trial of 156 adults, participants were assigned aerobic exercise, the antidepressant sertraline (Zoloft), or both [34]. When tested 4 months later, all three groups had a statistically significant improvement in depressive symptoms. Though the medication group had a faster response to treatment in the first 4 weeks, there was no statistically significant difference between the three groups at the 4-month time period. Similar results were reported in a follow-up study [35]. 

It appears that the exercise intervention need not be lengthy or intensive in order to be associated with a clinically significant reduction in reported symptoms of depression. For instance, in one study, 30 min of treadmill walking for 10 consecutive days was enough to produce a clinically and statistically relevant reduction in points from baseline on the Hamilton Rating Scale for Depression [36]. In addition, there was a significant correlation in subjective and objective changes in depression scores. However, evidence does indicate that less strenuous exercise may be comparable to placebo effects [37].

Though all or most forms of exercise appear to be beneficial for depressive symptoms, it appears that aerobic exercise may have an advantage over other forms of exercise [38]. In one study, participants placed in a placebo exercise group were required to do low intensity stretching and relaxation exercises. These participants had a smaller reduction in depression scores compared to the aerobic exercise group [39]. In addition, a larger proportion of patients exhibited a reduction of depressive symptoms in the exercise group compared to the placebo group (65% *vs.* 22%, respectively). 

Although these studies demonstrate that exercise can reduce symptoms of depression, the variable findings of these studies suggest future research should continue to explore the relationship between exercise and depression [40]. The fact that many individuals with depression may not be motivated to engage in exercise and would thus be screened out from the study may influence results. In addition, more studies using intent-to-treat analyses are needed to make findings more conclusive.

##### 1.2.1.2. Anxiety and Panic Disorders

As with patients with depression, individuals with anxiety and related disorders may benefit from exercise, though exercise has been less studied in this population (for review, see [41]). Furthermore, because anxiety disorders vary widely, it is difficult to generalize research conducted on one type of anxiety disorder to other anxiety disorders [30]. 

Research suggests that exercise may help reduce anxiety sensitivity. For instance, individuals with high scores on the Anxiety Sensitivity Index-Revised reported significantly less anxiety sensitivity following six 20-min aerobic exercise sessions [42]. Scores for self-reported fear of anxiety sensations, respiratory and cardiovascular symptoms, publically observable symptoms of anxiety, and cognitive dyscontrol all decreased in the exercise group. Scores for the no-exercise control condition, on the other hand, did not significantly change. 

Evidence suggests that as with depression, exercise may be just as beneficial for symptoms of anxiety as pharmacological treatment. In one study, 70 participants were randomly placed into one of four groups: cognitive group therapy, aerobic exercise, both treatments, or a control group [43]. Six weeks later, the change in the State-Trait Anxiety Inventory scores revealed that all three treatment groups were equally effective in reducing anxiety compared to the control group.

#### 1.2.2. Cognition

A large proportion of research on exercise and cognition in adult humans has focused on older adults. The neuroprotective effects of exercise have been of particular interest in this population. For example, in a longitudinal study of 349 participants over the age of 55, a positive correlation was reported between cardiovascular fitness and measures of global cognitive function [44]. Similar results were found in a longitudinal study that followed over 18,000 women between the ages of 70 and 81 for 9 years [45]. The women who had higher physical fitness levels not only had better performance on cognitive measures at the start of the study, but they also demonstrated less cognitive decline over the course of the study. 

Interestingly, the time in life at which exercise is initiated does not seem to be of great importance, though evidence does suggest that individuals who report having been physically active early in life tend to have less likelihood of cognitive impairment in older adulthood compared to those who report initiating exercise later in life [46,47]. The intensity of exercise, however, does appear to be important. Evidence suggests that at least moderate levels of physical activity are necessary to decrease the likelihood of cognitive decline in older adulthood [46,48].

As in patients with traumatic brain injury (TBI), the frontal, prefrontal, and temporal brain regions appear to be the most susceptible to the loss of brain tissue in older adults [49]. Importantly, losses in these regions are also correlated with cardiovascular fitness. As such, exercise appears to be most beneficial for frontally-mediated cognitive functions in older adults. In a randomized controlled study of 124 elderly participants, those who were placed in a walking intervention demonstrated significant improvement in executive functions such as planning, inhibition, and working memory [50]. The intervention did not significantly improve cognitive functions not related to the frontal lobe.

In a study that utilized fMRI, 29 high-functioning, community-dwelling older adults were randomly assigned to an aerobic fitness group or a stretching and toning control group [51]. After participating in the intervention for 45 min a day, 3 times per week for 6 months, the aerobic exercise group demonstrated a significantly higher VO_2max_. The same group had significantly lower reaction times on the Flanker task following the intervention, and fMRI data indicated that these participants had significantly increased activity in regions involved in attentional control, including the right medial frontal gyrus, superior frontal gyrus, and superior parietal lobe. The aerobic exercise group also demonstrated a significant decrease in activity of the anterior cingulate, which indicates that following the intervention, the participants had a decreased need to monitor conflict during complex cognitive tasks. 

Data from a similar study also indicates that exercise can increase brain volume in regions associated with age-related decline [52]. Fifty-nine healthy but sedentary community-dwelling adults between the ages of 60 to 79 were again randomly assigned to an aerobic exercise group or a stretching and toning control group. Gray and white matter volumes were collected using magnetic resonance imaging (MRI) before and after the 6-month long intervention. Results indicated that there were significant increases in both gray and white matter for the participants in the aerobic exercise group, but not for the control group. Importantly, this study demonstrates that aerobic exercise is capable of not only preventing decline in tissue volumes, but it might be used to recover some of the losses in brain volume incurred as a result of normal aging. 

The finding that exercise maintains and possibly increases brain tissue volume in older adults is important because there appears to be a relationship between cardiorespiratory fitness and brain atrophy in the earliest clinical stages of Alzheimer’s disease [53]. Early-stage Alzheimer’s participants with higher fitness levels had less brain atrophy. This was independent of age and dementia severity. This last factor is important because one common aspect of Alzheimer’s disease is that it is associated with reductions in physical activity, which in turn can reduce fitness levels. 

Evidence indicates that older individuals who already demonstrate cognitive impairments may benefit from exercise in the same way cognitively intact individuals do. For example, in a meta-analysis of thirty randomized trials, exercise was shown to increase physical fitness and function, cognitive function, and positive behavior in individuals with dementia and related cognitive impairments [54]. 

In addition, exercise may be able to prevent or postpone Alzheimer’s disease and other forms of dementia [55,56,57]. In a longitudinal study conducted on 4615 community-dwelling adults over the age of 65, the presence of physical activity at the beginning of the study was associated with lower risks of cognitive impairment, Alzheimer’s, and dementia of any type at the 5-year follow-up assessment [57]. In addition, a significant neuroprotective effect was found in women with the highest levels of physical activity. In another longitudinal study conducted on 2257 men between the ages of 71 to 93, the distance walked per day at the beginning of the study was linked to the probability of developing dementia up to eight years later [2]. 

#### 1.2.3. Neurological Damage

Emerging evidence in the human literature suggests that aerobic exercise may be therapeutic in individuals recovering from neurological damage caused by stroke or TBI. Cognitive deficits are observed in up to two-thirds of stroke patients, which can in turn negatively affect response to rehabilitation and global functioning [58]. Encouragingly, several studies have reported that aerobic exercise can improve both cognitive and motor functions in stroke survivors. In one study, nine older adults with chronic stroke participated in a 12-week exercise program [59]. The participants engaged in aerobic and lower extremity muscle strengthening exercise for three 1-h sessions a week. Post-test results indicated that there were significant improvements on the Digit Span Backwards test, a test of working memory; the memory component of the Stroke Impact Scale, a self-report questionnaire that asks about how life has changed after one’s stroke; and the Fugl-Meyer score, a test of voluntary motor control. Moreover, there was a significant correlation between improved aerobic capacity and improved performance on the Flanker test. 

In another study, 38 chronic stroke survivors were randomly assigned to either an aerobic exercise group or a stretching control group, which they participated in for 45 min a day, 3 times a week, for 8 weeks [60]. The aerobic exercise group had significantly improved VO_2max_ and information processing speeds based on performance on the serial reaction time task. This group also had significant improvement on sensorimotor control tasks. Interestingly, there was no significant improvement on tasks of executive function following the intervention, though Quaney and colleagues suggest that 8 weeks might not have been a long enough intervention to see cognitive effects. 

As with stroke patients, a large portion of individuals with TBI demonstrate impairments in cognitive function; one study reported that 33% of TBI patients had impaired cognitive function following discharge from the hospital [61]. Emerging evidence supports the claim that aerobic exercise may lead to cognitive improvement in TBI patients [62]. In one retrospective study, a community-based sample of 240 individuals was divided into an exercise or non-exercise group. Exercisers with TBI had fewer self-reported depression and cognitive symptoms [63]. 

In another study, 13 moderate TBI patients participated in a virtual reality aerobic exercise intervention for at least 25 min a day, 3 days a week, for 4 weeks [64]. The patients began the intervention at least 6 weeks after the injury. Following the intervention, the exercisers performed significantly better than age- and injury-matching controls on verbal, visual learning tasks, and processing speed tasks.

Thus, although evidence is still fairly limited, research suggests that aerobic exercise may have therapeutic benefits for cognitive performance in stroke and TBI patients.

## 2. Effect of Exercise on Behavior in Non-Human Animals

Physical activity may be defined in a multitude of ways. For some people, exercise is aerobic and includes activities such as walking, running and bicycling. For others, exercise is anaerobic and includes activities such as strength training, yoga and Pilates. Just as exercise varies across humans in real life, it varies in its application in non-human animal research. Whereas one study may examine the effects of a two-week exercise program on behavior, another may examine the extent to which behavior is affected after six months of physical activity. The following discussion describes how aerobic activity impacts animal models of affect and cognition. Due to the variability in how exercise is defined in this literature, the discussion is broken down to examine the extent to which short-term (less than three weeks of physical activity; acute) and longer-term (chronic) exercise contributes to behavior.

### 2.1. Acute Exercise

In the non-human animal literature, acute exercise is typically described as less than three weeks of physical activity. While most exercise paradigms utilize voluntary physical activity, namely in the form of wheel running, some paradigms also employ forced exercise through swimming or running on a motorized treadmill. The general consensus from this existing body of literature suggests that acute physical activity is capable of producing significant changes in a variety of behavior, and in the following section we will describe the ways in which it affects animal models of anxiety, depression, learning and memory. 

#### 2.1.1. Affect

##### 2.1.1.1. Anxiety

A classic measure of anxiety in non-human animal models is the open field test. First described by Hall in 1934 [65], the open field test is based on the observation that when provided access to a novel open environment, animals exhibit significantly less exploratory and locomotor behavior presumably due to a heightened level of anxiety over the new surroundings. The extent of exploration and locomotion is quantified by dividing the open field into several quadrants and measuring how many quadrant crossings occur, as well as how much time is spent in each quadrant. Animals that are more anxious tend to remain in, or around, the quadrant in which they are placed and therefore also spend a greater proportion of time there. Anxiety, as measured by the open field test, is altered in rodents that exercise acutely. Mello and colleagues [66] found that rats subject to two weeks of forced treadmill running exhibited substantially more locomotor behavior relative to inactive controls. Similarly, Salam and colleagues [67] demonstrated that after two weeks of voluntary wheel running mice spent more time in the center of the open field, made more crossings between the quadrants of the open field and made fewer escape attempts when compared to sedentary cohorts. The length of the open field test may in itself affect anxiety, however. In contrast to observing a beneficial effect of voluntary exercise on anxiety when observed for six minutes in an open maze, as was done in the Salam *et al.* [67] study, Hopkins and Bucci [68] reported an increase in anxiety, as measured by significantly less locomotion, in rats that were exposed to an open field for 15 min. This finding is particularly interesting given that both experiments reported identical exercise regimens. Given that vast behavioral differences exist between rats and mice [69], as well as within rat [70] and mouse strains [71], a possibility for this inconsistency is that animal species interact with exercise such that mice are less susceptible than rats to anxiety in a novel, open environment. 

In addition to the open field test, anxiety in non-human animal models may be assessed by measuring their response to the presentation of unexpected and sudden stimuli. Under normal conditions, such stimuli elicit a startle response characterized by an intense behavioral response (startle), followed by a period of apparent immobility (freezing). Both the amplitude of the startle response and proportion of time spent freezing are positively related to anxiety. In two separate studies, mice were acclimated to a sound attenuating chamber for one day after two weeks of free access to a running wheel. Following the acclimation period, several bursts of white noise were presented in a pseudo-random fashion and the startle amplitude was recorded. The results indicated that the mean startle amplitude was significantly lower in the mice that had exercised compared to sedentary controls [67,72]. Bursts of intense visual stimuli have also been shown to produce startle in rodents, which is attenuated by acute physical activity [67]. 

Taken together the current literature suggests that short-term exercise regimens help protect against anxiety produced by unfamiliar contexts and environmental stimuli. This benefit appears to be achieved by exercise in general rather than exercise of a particular nature (*i.e.*, voluntary, forced). That said, physical activity might be explored as a method used to jump-start the treatment of pathological fear in humans. More research in this area is certainly needed, however, to determine whether such an option is viable. 

##### 2.1.1.2. Depression

Like anxiety, the ways in which depressive symptomology manifest in non-human animals is very similar to humans. For example, both models tend to exhibit anhedonia, which is characterized by a profound decrease in typically pleasurable activities, as well as learned helplessness. A search of the literature revealed that there has been no examination of the relationship between acute (less than three weeks) physical activity and depression in non-human animal models. This lack of information requires immediate attention. 

#### 2.1.2. Cognition: Learning and Memory

The ability to acquire new information and store it long term has been studied extensively. A vast body of literature supports the idea that memory is generally divided into two forms. Explicit, or declarative memory, refers to one’s memory for factual information such as who is the current president of the United States and what one ate for breakfast. In contrast, implicit, or non-declarative memory, describes one’s knowledge of things not so easily described such as how one achieves balance when riding a bike. While the ability to “declare” knowledge suggests it requires language, measuring declarative learning and memory can be achieved through simple behavioral observation. The measurement of implicit information can also be quantified behaviorally. Using non-human animal models to examine the extent to which both explicit or implicit learning and memory occurs is therefore relatively simple. The following discussion will illustrate how learning and memory is measured by animals’ performance on mazes and other classic paradigms, and how each process is mediated by physical activity. 

Classical conditioning is a form of associative learning and memory that occurs when a neutral conditioned stimulus (CS; e.g., tone or light) is repeatedly presented with an unconditioned stimulus (UCS, e.g., foot shock or food) such that after repeated pairings, the organism anticipates the UCS whenever presented with the CS. There are a number of ways to measure the strength of the associative relationship between CS and UCS in non-human animals. During auditory-cued fear conditioning, an animal is placed in a sound-attenuating chamber where it is presented several trials consisting of a tone CS followed by a mild footshock UCS. A test trial is administered after this acquisition phase in which the tone CS is presented in the absence of the footshock UCS. The amount of freezing is measured and provides an index of learning and memory. Not surprisingly, under normal conditions, the percent of time freezing is much greater during the test phase than during the initial trials of the acquisition phase. A number of factors, including acute physical activity, have been shown to affect the acquisition and retention of auditory-cued fear conditioning [73]. In addition to learning that the tone predicts shock, the environment (context) in which the shock is delivered also leads to a conditioned fear response. Indeed, two weeks of free access to a running wheel enhances contextual fear conditioning in rats, with the most notable difference between exercised and sedentary animals occurring during the early portions of the acquisition phase [68]. Acute voluntary wheel running has most recently been shown to enhance eyeblink conditioning in rats. Compared to sedentary controls, rats permitted free access to a running wheel for 17 days exhibited a shorter response latency to a tone CS that preceded an ocular airpuff UCS [74]. 

In addition to classical conditioning, learning and memory in non-human animal models has been assessed using a variety of mazes, one of which is the Morris water maze [75]. The Morris water maze (MWM) assesses spatial learning and memory through the observation of behavior exhibited by animals that have been placed into a pool that contains an escape platform. Because the platform is hidden by opaque water, animals must rely solely on extra-maze cues to find its location and escape the water. Acute physical activity is shown to improve MWM performance in rodents. Ten days of voluntary exercise enhanced both acquisition and retention of the hidden platform in the MWM [76] and Vaynman and colleagues [77] observed improved MWM performance in animals that had exercised only five days before training. Furthermore, in another test of spatial memory, the Y-maze, animals provided comparable levels of physical activity demonstrated enhanced acquisition and memory retention of the task [78]. 

While there is evidence that short-term exercise programs improve learning and memory in non-human animals, it should be noted that not all studies of acute exercise yield positive results. O’Callaghan and colleagues [79] report that seven days of forced exercise are sufficient to improve object recognition performance. However, O’Callaghan and colleagues did not observe improved MWM performance following seven days of exercise. Similarly, Mello and colleagues [66] found that sedentary controls took about the same time to find the platform in the MWM as animals that had voluntarily exercised for two weeks. There are several explanations for the inconsistent findings regarding voluntary exercise and cognitive performance. For instance, particularly in MWM testing, there are a variety of protocols used to assess learning performance and some of these protocols may be more sensitive than others in detecting cognitive facilitation [80]. It is also possible that some of these findings are due to inherent differences in voluntary and forced exercise paradigms and may reflect the effects of stress on learning and memory performance. Forced exercise is a stressful manipulation that increases anxiety-like behaviors in rodents as measured in the open field [81] and is associated with symptoms commonly observed with chronic activation of the hypothalamic-pituitary-axis (HPA) including adrenal hypertrophy, thymic involution and suppressed lymphocyte proliferation, indicating that there may be negative effects of forced treadmill running [82]. These effects of forced exercise may limit its ability to improve learning and memory function in some assessment paradigms.

### 2.2. Chronic Exercise

#### 2.2.1. Affect

##### 2.2.1.1. Anxiety

Whereas acute exercise tends to reduce anxiety in non-human animal models, the relationship between chronic physical activity and anxiety is less clear. For instance, whereas one study found that rats forced to exercise for eight weeks did not differ from sedentary controls with regard to locomotion in an open field test [66] another reported that eight weeks of forced exercise significantly increased anxiety using the same measure [81]. Contradictory evidence also exists when chronic exercise is voluntary in nature. Whereas three or four weeks of free access to a running wheel significantly reduced anxiety in mice on the open field test [83,84,85], more prolonged voluntary exercise regimens produced an increase in anxiety on the elevated plus maze [86]. 

Together this body of literature suggests that the relationship between chronic exercise and anxiety in non-human animals is ambiguous, most likely because of the inconsistency in how exercise is operationalized. However, based upon what is currently known, it appears that in order to protect against anxiety, exercise should be voluntary in nature and performed over a relatively short period of time. 

##### 2.2.1.2. Depression

In contrast to the lack of information with regard to whether acute exercise affects depressive symptomology in non-human animal models, a wide body of literature suggests that chronic physical activity has a positive effect on affect. Pharmacologically-induced depression was prevented in animals forced to swim daily for three weeks. Compared to their sedentary cohorts, swimmers also consumed more sucrose-flavored water and exhibited a greater degree of mobility in the pool [87] both of which are indices of normal affect. The consumption of sucrose-flavored water was also measured in rats exposed to four weeks of unpredictable stress concomitant with free access to running wheels. The results indicated that compared to non-stressed cohorts, rats that received chronic stress consumed significantly less of the flavored water. When rats were provided with free access to running wheels during the stress paradigm, anhedonia was significantly attenuated suggesting that at least four weeks of voluntary physical activity is sufficient to positively affect mood, even in the presence of less-than-optimal environmental conditions. 

In addition to anhedonia, chronic inescapable stress also produces learned helplessness in non-human animal models. In rats, the latency to attempt to escape electric shock in a shuttle box is an index of learned helplessness. Twenty-four hours after receiving four to twelve weeks of voluntary exercise, rats were placed in a shuttle box and escape latency was measured. The results clearly demonstrate that animals permitted physical activity make an immediate attempt to escape shock compared to their sedentary cohorts [88,89].

#### 2.2.2. Cognition: Learning and Memory

It is well established that chronic exercise also improves learning and memory in non-human animal models. Rats exposed to stress that were also provided free access to running wheels for four weeks exhibited enhanced learning of the MWM. Compared to un-exercised cohorts, stressed rats that exercised had a significantly shorter swim latency to platform. The benefit of exercise on learning is strengthened by the observation that stressed-exercise rats performed comparably to exercise-only rats, and better than unexercised-nonstressed rats [85]. In addition to stress, age affects spatial learning such that older animals perform poorly on the MWM relative to young animals [90]. In mice, the negative effect of ageing on spatial learning was blocked by four weeks of chronic voluntary exercise such that the mean swim latency to platform was equivocal in aged runners and young runners [90]. Some studies suggest that physical activity does not affect spatial learning, however. Rhodes *et al.* [91] reported that active and inactive mice performed comparably on the MWM. It is important to note, however, that the mice used in this study were specifically bred for increased running making it possible that the genetic manipulations may have contributed to the null result. Overall, the research indicates that in normal animals, physical activity is beneficial to spatial learning.

There is some evidence to suggest that the nature of exercise does not affect the degree to which learning and memory occur in the MWM. Rats forced to exercise (FX) on a treadmill, as well as rats allowed to exercise voluntarily on wheels (VX), for four weeks exhibited more rapid learning and greater memory retention of the hidden platform when compared to sedentary controls [92]. Leasure and Jones [81] also found that FX and VX rats were equal in terms of MWM escape latency and time spent in the correct quadrant. However, when the VX and FX rats were trained on a one-trial passive avoidance test, only the FX rats performed better relative to controls [92]. 

## 3. Cellular Mechanism of Action

The mechanisms by which experience (including exercise, enriched environments, and motor skill learning) facilitates learning and memory in non-human animals are not fully understood. However, there are certain physiological effects associated with general experience that have been shown to contribute to enhanced cognitive performance, including structural changes such as synaptogenesis (the development of new synapses), angiogenesis, and neurogenesis [90,93,94,95,96,97,98,99,100]. Experience also results in molecular processes such as the upregulation of specific growth factors associated with these structural changes, including IGF-1, BDNF, and VEGF [101,102,103,104,105,106,107,108,109]. For the purpose of this review, we will focus on aerobic exercise as a particular experience that is most associated with angiogenesis and neurogenesis as well as various growth factors associated with each of these processes.

### 3.1. Neurogenesis

One type of experience-induced plasticity following exercise involves the development of new neurons, a process known as neurogenesis. Originally believed to be exclusive to development, neurogenesis has been detected in adult mammals including rodents, non-human primates [110,111,112,113,114], and humans [115]. This process is regionally specific and occurs in the subventricular and subgranular zones of the dentate gyrus of the hippocampus as well as in the olfactory bulb [110,113,116]. Some of the new neurons in the hippocampus and olfactory bulb replace dead or dying neurons in the system, while others appear to support new learning functions [117,118].

The survival of new neurons may contribute to learning and memory changes following exercise. It has been consistently shown that both enriched environments and exercise (voluntary and forced) promote neurogenesis in the adult hippocampus, specifically in the dentate gyrus [90,96,98,99,119,120,121,122,123]. Kim *et al.* [119] report increased Bromodeoxyuridine (a mitotic cell marker; BrdU) labeling following as little as 30 minutes of forced exercise for seven consecutive days. Exercise-induced neurogenesis occurs even in aged animals. Increased neuronal labeling has been observed in exercised mice that are as old as 24 months of age at the cessation of the running regimen [124]. The onset of exercise-induced neurogenesis is rapid, and is detectable after only 12 h of voluntary exercise in rats and persists for at least one week into the exercise regimen [125]. The means by which exercise increases neuronal labeling appears to be related to elevated levels of cellular proliferation [121]. Research has shown that exercise is associated with the upregulation of growth factors including IGF-1, FGF-2, and BDNF, which may be responsible for increased cellular proliferation in exercising animals and will be discussed further elsewhere in this review. Additionally, blood flow (and consequently metabolic support) is increased with exercise, which also may contribute to enhanced cellular proliferation in exercising animals [121,126,127]. 

Exercise-induced neurogenesis has frequently been correlated with improved learning and memory performance. Following eight weeks of forced treadmill running, young male rats (22 days of age at the beginning of the exercise regimen) exhibited increased numbers of hippocampal CA1 and CA3 neurons in addition to an increased number of dentate gyrus neurons as well as superior MWM performance [121]. These findings indicate that exercise promoted neurogenesis and neuronal survival in the hippocampus that was correlated with facilitated learning and memory performance. Similar results have been reported in aged mice (19 months at exercise onset), with exercised mice expressing levels of neurogenesis and cognitive performance similar to those in young exercised animals [91]. 

While there is an association between neurogenesis and enhanced cognitive performance following exercise, the necessity of neurogenesis to cognitive facilitation has not been fully explored and currently there is somewhat contradictory evidence regarding this issue. It has been reported that neurogenesis is not required for the enhancing effects of enriched environment housing on MWM performance [128]. Mice received focal X-irradiation of the hippocampus, which selectively prevents hippocampal neurogenesis [129], prior to a six-week exposure to an enriched environment that included free access to running wheels. Meshi and colleagues [127] found that all enriched animals, regardless of X-irradiation treatment, exhibited improved performance in the MWM evidenced by decreased total distance traveled, decreased latency to find the hidden platform, and increased proportion of trial time spent in the target quadrant. Additionally, X-irradiation had no effect on anxiolytic behavior following enriched environment exposure. These results indicate that neurogenesis is not a critical factor in the cognitive benefits of an enriched environment.

Clark and colleagues [129] reported that intact neurogenesis is required for some, but not all, of the cognitive benefits of voluntary exercise. Mice were permitted to voluntarily exercise on running wheels for a period of 54 days following X-irradiation of the hippocampus. Histological analysis confirmed that exercise increased neurogenesis approximately fourfold, while radiation reduced neurogenesis by 50%. Exercise improved MWM performance only in sham-irradiated mice. X-irradiated mice exhibited no cognitive benefit of exercise in the MWM. However, X-irradiation did not affect all cognitive facilitation as both sham- and X-irradiated animals exhibited similar increases in freezing in a contextual fear conditioning paradigm compared to inactive controls. 

Differences between Meshi *et al.* [127] and Clark *et al.*’s [129] findings may be due to inherent differences in enriched environments and voluntary exercise. As reviewed by Olson and colleagues [120] the two paradigms promote neurogenesis through dissociable pathways, with exposure to enriched environments preferentially affecting neuronal survivability and voluntary exercise promoting cellular proliferation. Therefore, improved cellular survivability in the Meshi *et al.* [127] study may account for facilitated MWM performance. It should be noted that mice in the Meshi *et al.* [127] study did have access to running wheels, although the extent to which animals engaged in voluntary running was not quantified. It is also possible that differences in methodology account for the detection of cognitive facilitation in one study but not the other. Some MWM protocols may better detect subtle differences in hippocampal function than others. The inconsistent data indicate that these processes have not been fully explored and further research regarding the mechanisms underlying experience-induced cognitive facilitation is necessary.

There are also some contradictory reports regarding the functional significance of adult neurogenesis that are not easily explained. Shors and colleagues [130] report that treatment with the antimitotic agent, methylazoxymethanol acetate (MAM), resulted in decreased neurogenesis without affecting MWM latency performance. MAM treatment did not affect contextual fear conditioning, another task commonly believed to require intact hippocampal circuitry. However, neurogenesis was found to be required for some learning paradigms that preferentially engage the hippocampus, specifically trace fear conditioning [130,131]. Shors and colleagues suggest that perhaps spatial learning does not require new neurons to facilitate task performance. It is also possible that MAM treatment may affect additional physiological processes that impact some but not all hippocampally-engaged memories. 

Results from experiments involving MAM inhibition of neurogenesis certainly suggest that adult neurogenesis serves an important role in some types of learning and memory. However, these results are somewhat complicated. Recent evidence indicates that while MAM is effective in blocking adult-onset neurogenesis, it has rather nonspecific effects. MAM also reduces exercise-induced angiogenesis [132], indicating that MAM may adversely affect cellular mitosis in general and lack the specificity to preferentially eliminate neurogenesis. 

The contribution of neurogenesis to learning and memory function is further complicated by recent evidence suggesting that newly proliferated neurons are not immediately and functionally incorporated into existing learning networks. While it is clear that some new neurons do become functionally integrated into the existing circuitry eventually, several reports indicate that this integration is a somewhat delayed process taking between three and four weeks to complete [133,134,135,136]. These new neurons are preferentially integrated into these circuits when they are between four to eight weeks of age [133]. Further, new neurons are as much as twice as likely to be recruited into spatial memory networks six to eight weeks following proliferation. These data are supported by behavioral studies indicating that impaired neurogenesis does not affect visual spatial memory in the MWM immediately following treatment but impairs performance when memory is tested 28 days later [136]. Anatomical data regarding neuronal development and synapse formation in the hippocampus support these findings. Hastings and Gould [137] found that while immature granule cells began to extend axons into CA3 between four and ten days following mitosis, these cells did not express a mature neuronal phenotype until approximately three weeks after BrdU labeling. Additionally, newly generated cells did not express dendritic spines until several weeks (15–17 days) following mitosis. Peak spine growth has been detected around three to four weeks after generation [138], indicating that these cells do not make significant synaptic connections until this time.

### 3.2. Angiogenesis

Angiogenesis (the sprouting of new blood vessels from pre-existing capillaries) is an important physiological process in development as well as in pathological conditions such as tumor growth [139,140,141,142,143,144,145]. Although initially believed to be an exclusively developmental phenomenon, non-pathological angiogenesis is now understood to occur in adult animals in response to experience, including both exercise and exposure to enriched environments [95,97,100,146,147,148]. 

Swain and colleagues [100] found that thirty days of exposure to running wheels resulted in increased cerebral blood volume accompanied by increased capillary volume in the motor cortex and increased immunolabeling of the α_v_β_3_ (CD61) integrin, which is expressed predominately on developing capillaries. Increased blood vessel density has been shown to occur in the absence of alterations in cortical movement representations in the motor cortex [147]. 

Similar results have also been reported in the cerebellum, with a peak in exercise-induced angiogenesis observed within three days of exercise onset [124,149]. Kerr and Swain [124] found that levels of the α_v_β_3_ integrin in the cerebellum increased within the first twenty-four hours of voluntary exercise and remain elevated for at least one week. Angiogenesis in the adult cerebellum has also been reported with 30 days of repetitive exercise. Quantified as diffusion distance from blood vessels in the molecular layer of the cerebellar paramedian lobule, angiogenesis is increased in forced exercise and voluntary exercise relative to both inactive controls and animals exposed to the acrobat task [97].

Exercise also increases blood flow, vascular density, and angiogenesis in the hippocampus [124,132,150]. Much like in the cerebellum, levels of angiogenesis (measured via levels of CD61) increase rapidly in the hippocampus (within two days of exercise onset) and remain elevated for about one week [124]. In addition, these increased levels of vascular labeling can be detected one month after exercise onset [132], indicating that early angiogenesis following exercise onset results in a relatively permanent increase in vascular flow in the motor cortex, cerebellum and hippocampus (at least as long as the aerobic activity continues). Therefore, aerobic exercise results in global angiogenesis that occurs rapidly following exercise onset; a plastic change that alters blood flow and metabolic support to surrounding tissues for the long-term.

Kerr and colleagues [126] found that angiogenesis, and not neurogenesis is required for improved MWM performance following exercise. Using the neurogenesis inhibitor AZT (100 mg/kg) and the angiogenesis inhibitor SU5416, researchers were able to selectively inhibit either neurogenesis or angiogenesis in animals that received free wheel access for 7 days prior to MWM training. Rats that received SU5416 exhibited the poorest MWM performance, while animals that received AZT displayed improved MWM performance compared to SU5416-treated and control animals. The findings of this study indicate that angiogenesis is a critical component of exercise-induced cognitive facilitation. Further, because new neurons are not functionally integrated into visual spatial networks following only seven days, these results also suggest that the rapid onset of neurogenesis following exercise may result in competition between new and existing neurons that somewhat complicates learning and memory performance, resulting in a baseline performance that is somewhat worse than in animals without neurogenesis.

It appears as though the connection between neurogenesis and angiogenesis may be a functional relationship whereby neurogenesis requires angiogenesis. Data suggest that long-term inhibition of angiogenesis prevents exercise-induced neurogenesis. Daily administration of the polyunsaturated fatty acid, Conjugated Linoleic Acid (CLA) resulted in decreased BrdU labeling in the hippocampus of exercised animals. Interestingly, CLA administration resulted in similar levels of BrdU labeling as MAM administration, a drug previously shown to inhibit neurogenesis. Both CLA- and MAM-treated rats expressed BrdU labeling similar to inactive controls [132]. Further, the time course of angiogenesis and neurogenesis following exercise onset is somewhat related. As explained previously, increased levels of neurogenesis in the hippocampus can be detected almost immediately (within 12 h) following exercise onset. Levels of angiogenesis peak within 48 h of exercise onset and persist as long as levels of neurogenesis remain elevated [124]. This time course suggests that angiogenesis may occur in response to increased neurogenesis in an effort to provide appropriate levels of metabolic support for newly established neurons to differentiate, survive, and integrate into the existent neural circuitry. 

### 3.3. Apoptosis

An interesting consequence of acute aerobic exercise is apoptosis, or programmed cell death. While levels of apoptosis are not found to be elevated after long-term exercise regimens [121,151], Kerr and Swain [124] report that, in the very short term, exercise is associated with central nervous system apoptosis, measured by levels of cleaved caspase-3 in both the hippocampus and cerebellum. Elevated levels of caspase-3 can be detected in both structures within 12 h of exercise onset, peaking after 24 h of exercise, and declining steadily over time. In fact, increased levels of caspase-3 are resolved by 48 h following exercise onset, which is the same time at which levels of angiogenesis increase. The timing of these processes suggests that apoptosis may promote the plastic changes commonly reported to occur with extended periods of exercise. 

The most parsimonious explanation for the increased cell death observed by Kerr and Swain [124] is that exercise increases the metabolic demand (resulting in improper metabolic supply) on these structures and the nervous system simply cannot accommodate the increased demand adequately. Increased cellular activity is associated with increased energy metabolism in the brain [152], and voluntary wheel running has been found to increase activity in the hippocampus [153]. Previous research indicates that exercise may result in changes in the brain that are consistent with a response to oxidative stress, including the generation of reactive oxygen species and consequent antioxidants [154,155,156,157]. It is possible that exercise induces mild hypoxia in the CNS, which results in elevated cell death. This hypothesis is supported by the fact that caspase-3 activity has been implicated as a potential mechanism of hypoxia-induced apoptosis [158]. As such, it can be hypothesized that increased demand for glucose and oxygen that occurs with exercise onset results in this apoptosis, as exercise is known to impact glucose transport in the cerebellum [159] and tissue oxygenation in skeletal muscle [160,161,162]. Therefore, when the animal is exposed to aerobic activity, those cells that have insufficient resources (because they require additional metabolic availability) to support the increased activity associated with exercise, undergo apoptosis.

## 4. Molecular Mechanisms

As previously described, exercise results in significant changes in brain anatomy and morphology. Specifically, exercise has been consistently shown to induce angiogenesis and neurogenesis in the brain, and can even regulate normal processes of programmed cell death [119,121,124,151,163]. Angiogenesis, neurogenesis, and apoptosis are all processes that are known to be controlled by several different molecules, some of which change in expression patterns in response to exercise. Most of these exercise-affected molecules fall into the category of neurotrophic factors [164], which are substances that promote the growth or survival of neurons by binding to their appropriate receptors on target cells and cell populations [164,165]. Some growth factors and receptors that are altered in response to exercise include vascular endothelial growth factor (VEGF) [166,167,168,169], brain-derived neurotrophic factor (BDNF) [141,170,171,172], nerve growth factor (NGF) [104,107], and insulin-like growth factor 1 (IGF-1) [173]. Several of these molecules have also been implicated in exercise-induced improvements of cognitive function [68,170,171].

### 4.1. Vascular Endothelial Growth Factor (VEGF)

VEGF is a growth factor that causes angiogenesis in both normal physiological conditions, such as the development of the corpus luteum during menstruation [174,175,176], and in pathological conditions such as exposure to hypoxia, or conditions of low oxygen tension [177], wound healing [178,179], and tumor growth and survival [145,180,181]. Furthermore, application of VEGF to brain slices *in vitro* or infusion of VEGF intravenously in an *in vivo* model results in significant increases in the vascularity of the brain [182]. 

Exercise itself has also been shown to upregulate VEGF expression and plays a vital role in exercise-induced angiogenesis [166,167,168,169]. Several studies show that both VEGF mRNA and VEGF protein are upregulated in various brain regions of animals exposed to exercise, and this increase in VEGF expression is often accompanied by an increase in blood vessel density in the brains of exercising animals [166,167,169]. Tang and colleagues [169] identified increases in VEGF mRNA and protein in the hippocampi of mice following one hour of intense treadmill exercise. In addition, longer term exercise resulted in significant increases in blood vessel density in the striatum and a trend toward increased blood vessel density in the cortex of animals that had exercised on a treadmill for three weeks [104]. In a later study, these authors also found increases in both VEGF mRNA and protein, along with significant increases in blood vessel density in both the cortex and striatum of aged animals exposed to exercise [166]. These findings indicate that VEGF is involved in exercise-induced angiogenesis, and additional research has gone further and investigated the impact of exercise on other proteins that are also involved in exercise-induced increases of VEGF expression.

Along with increases in VEGF activity it would follow that the expression of receptors for VEGF would also be increased; without successful binding to its receptors, VEGF would fail to have a significant effect on blood vessel density in response to exercise. The two high-affinity VEGF receptors, flk-1 and flt-1, have somewhat different functions. The flk-1 receptor has been shown to be vital in the formation of the vasculature during embryogenesis, as mice lacking this receptor die *in utero* due to a failure to form an adequate vascular supply system [183,184]. In mature animals under normal conditions, flk-1 receptor expression is significantly downregulated compared to its expression in developing embryos [185]. Contrastingly, the flt-1 receptor is not necessarily responsible for the formation of vasculature but rather in the organization of the vasculature [186]. Like the flk-1 receptor, the flt-1 receptor is present during embryogenesis but unlike flk-1 it is present in large quantities in adult tissues [187], indicating that flt-1 may act to continuously maintain vascular organization [141]. It has been suggested that flt-1 serves as a “stop signal” for angiogenesis during embryonic development, which prevents the vasculature from overgrowth and organizes the successfully formed blood vessels into a cohesive network [184,186]. Overall, all of these findings suggest that whereas flk-1 is more involved in the actual process of the formation of blood vessels, flt-1 is more important for the organization of the newly formed network of vasculature. As such, along with increased expression of VEGF, increased expression of both flk-1 and flt-1 would be expected in response to exercise—flk-1 to be involved in the formation of new capillaries and flt-1 to successfully organize the newly formed vasculature. 

Specific research regarding the effect of exercise on the expression of the VEGF receptors has been conducted and has identified significant increases in these receptors [159,188]. Just as VEGF is expressed in both the periphery and the brain in response to exercise, the high-affinity VEGF receptors are significantly upregulated in response to exercise training in both peripheral [189,190,191,192] and nervous tissues [159,188]. Lloyd *et al.* [190] found significant increases in both muscle capillarity and in the expression of VEGF mRNA in skeletal muscles of rats soon after the commencement of a treadmill exercise training regimen. In addition to these findings, they also assessed the expression of flk-1 and flt-1 in the muscles and the timelines along which their expressions increased. Their data showed significant increases in both flt-1 and flk-1 following just one day of training and declining to near baseline levels around day nine; interestingly, both receptors were upregulated and returned to baseline levels at approximately the same time. These upregulations in VEGF receptors coincided partially with increases in VEGF mRNA (VEGF mRNA remained elevated until day 12), and preceded significant changes in capillary density, which makes sense as the signaling mechanisms needed to be in place before morphological changes to the vasculature could be observed [190]. 

In a recapitulation of the findings regarding the activity of the VEGF receptors in response to exercise in the periphery, studies have identified similar changes with regard to VEGF receptor expression in the brains of exercising animals. Thompson and colleagues [159] allowed animals free access to a running wheel for 0, 2, 4, 10, or 30 days and found significantly increased expression of flk-1 receptors in the cerebellum (paramedian lobule; representing the rat forelimb) of animals that had exercised for just two days. However, flk-1 expression was not significantly different than controls at any other time point. More interestingly is the additional finding that flt-1 labeling was significantly decreased at the two-day time point. These findings further highlight the significant differences between the two high-affinity VEGF receptors, and support previous research that show that whereas flk-1 is important for neovascularization, flt-1 is involved in the organizational functionality of newly formed capillaries from a preexisting network. Furthermore, the time at which flk-1 expression was increased coincides with the increase in exercise-induced angiogenesis described by Kerr and Swain [124]. 

In addition to the cerebellum, increased flk-1 expression has also been identified in the motor cortex of exercising animals, but this increase is delayed relative to the increase of flk-1 labeling in the cerebellum [188] as a significant increase was not observed until animals had exercised for 10 days. It is unclear why the exercise-induced alteration of VEGF receptor expression is delayed in the motor cortex compared to the cerebellum, but it may suggest a recapitulation of developmental processes in which more posterior brain structures develop first. Although VEGF and its high-affinity receptors are most often discussed with regard to their relation to exercise-induced angiogenesis, some research indicates that VEGF and the VEGF receptors contribute to other exercise-induced changes to brain anatomy.

Activity-induced neurogenesis also appears to require the activity of VEGF. Fabel and colleagues [105] found that blocking peripherally produced VEGF by way of an adenovirus targeting one of the high-affinity VEGF receptors (flt-1), totally abolished exercise-induced cell proliferation in animals allowed access to running wheels for seven days. Interestingly, they also noted that running did not significantly increase VEGF in the hippocampus itself. The authors argue that the absence of changes in hippocampally produced VEGF suggests that either hippocampal VEGF fluctuations are too small to detect, or that it is only the VEGF that is produced in the periphery that is responsible for exercise-induced neurogenesis. Their findings seem to support the latter argument, which further implies that even molecular pathways located outside of the brain contribute to significant neural changes in response to exercise. Indeed, several of the same mechanisms that lead to angiogenesis and increased blood flow in the brain in response to exercise have also been identified in the periphery, particularly in skeletal muscle, where VEGF protein, VEGF mRNA, and capillary density have been found to be significantly increased in response to exercise [192,193].

### 4.2. Brain Derived Neurotrophic Factor (BDNF)

Another notable growth factor that has been consistently shown to increase in the brain in response to exercise is brain derived neurotrophic factor (BDNF) [107,170,172,194,195,196]. In general, BDNF promotes the survival of central nervous system neurons [197] and also affects the outgrowth of neurons *in vitro* [198]. BDNF is abundantly expressed in the hippocampus, and although research has found that it is not highly expressed during developmental processes, it is expressed in large amounts in mature neurons [199], highlighting its role in neuronal survival. Therefore, it is not surprising that BDNF has become a molecule of interest in the exercise research literature. 

Neeper and colleagues [107] allowed rats access to a running wheel for two, four, or seven nights and found significant increases in BDNF mRNA in several regions of the brain compared to control animals. More specifically by region, animals training for two, four, or seven days had significantly greater levels of BDNF mRNA in both the hippocampus and caudal cortex compared to sedentary controls; BDNF mRNA levels were significantly higher in cerebellum following four and seven days of running; the frontal cortex BDNF mRNA expression was increased following two days of voluntary exercise. To determine the timeline of BDNF mRNA expression following exercise, Rasmussen *et al.* [195] exposed mice to an acute bout of treadmill exercise and found significant increases in hippocampal and cortical BDNF mRNA levels, with levels peaking at 2 h post-exercise and returning to baseline levels by 24 h after exercise cessation. 

Long-term exercise paradigms also induce significant changes in BDNF expression. In another study, BDNF protein measurements were made rather than mRNA measurements, and significant increases in protein were not identified until day 28 of voluntary exercise [200]. These findings are consistent with additional BDNF protein findings by Berchtold and colleagues [170], who found significant increases in the hippocampus of mice allowed running wheel access for three weeks. Therefore, it appears that changes in BDNF mRNA may occur much more rapidly following exposure to exercise compared to BDNF protein changes. Furthermore, and in contrast to BDNF mRNA expression which returns to baseline levels just 24 h after exercise cessation [195], increases in BDNF protein levels were found to persist up to two weeks after exercise cessation [170]. However, more prolonged periods of inactivity ultimately result in a decrease of BDNF protein to baseline levels, as noted by Radak *et al.* [172] who identified increases in BDNF protein following eight weeks of swimming training, but these changes in neurotrophin levels were no longer present after eight weeks of detraining (being sedentary). These findings indicate that while BDNF increases may persist for some time after exercise cessation, prolonged periods of inactivity still result in decreases back to baseline. Overall, findings of increased BDNF mRNA and protein in the brains of exercising animals, and the timelines of expression increases and decreases suggests that maintenance of an exercise regimen could result in prolonged upregulation of BDNF which could result in significant changes to the structure of the brain and brain function. 

Given that exercise increases BDNF activity and causes angiogenesis, and in considering evidence that BDNF serves as an angiogenic factor in some cancers [201], it is reasonable to hypothesize that BDNF may also be involved in exercise-induced angiogenesis. Ding and colleagues [104] reported that after three weeks of treadmill exercise, rats had significantly greater expression of BDNF in the cortex and striatum compared to sedentary animals. Furthermore, significant increases in blood vessel density were also found in the striatum, with a trend toward significantly increased blood vessel density in the cortex. The concomitant increases in both BDNF expression and angiogenesis suggest that BDNF may play a role in exercise-induced angiogenesis; however few studies have examined the direct relationship between this neurotrophic factor and angiogenesis. 

The relationship among exercise, BDNF expression, and neurogenesis has also been investigated. For example, Farmer and colleagues [202] identified significant increases in the number of neurons and in BDNF mRNA in the dentate gyrus of animals allowed access to a running wheel. Additionally, Kim and colleagues [151] exposed both young and aged rats to treadmill exercise and found that after six weeks of training, both young and old animals had significantly greater volumes of proliferating neurons in the dentate gyrus of the hippocampus compared to age-matched sedentary animals. Furthermore, exercising animals from both age groups had significantly higher levels of BDNF protein in the dentate gyrus compared to control rats. Therefore, as with the findings of exercise-induced increases in angiogenesis occurring alongside increases in BDNF, the fact that neurogenesis increases as BDNF also increases following exercise, suggests that the process somehow involves the actions of BDNF. As mentioned previously, BDNF is known most prominently for its role in neural survival. Therefore, even if BDNF is not directly involved in the actual birth of new neurons, it is involved in the survival of those neurons as demonstrated by increased numbers of proliferating cells. 

### 4.3. Nerve Growth Factor (NGF)

Nerve growth factor (NGF) was the first neurotrophic factor to be identified by Levi-Montalcini and Cohen [203], who found that addition of a substance, later isolated and defined as NGF, to the ganglia of chick embryos caused dramatic growth of nerve fibers from the ganglia. In addition to the effects of NGF on neurogenesis, other studies have noted that administration of NGF can also significantly increase capillary density. For example, Romon *et al.* [204] found that endogenous NGF in immunocompromised mice caused significant angiogenesis in implanted breast cancer cells. Additionally, administration of NGF caused angiogenesis in the chick embryo [205], in ischemic hindlimbs of mice [206], and in rat corneas [207]. 

Research has also found increases in NGF in several areas of the brain in response to exercise [104,107,196]. Zhu *et al.* [196] found that socially housed animals that were allowed 24-h access to a running wheel on alternating days for six weeks had significantly greater NGF protein levels in the hippocampus and frontal cortex compared to animals exposed only to social housing. Similarly, in another study, increases in NGF mRNA were observed in the hippocampus after two days of wheel running, and in the caudal cortex at two, four, and seven days of wheel running [107]. Finally, Ding and colleagues [104] found significantly increased levels of NGF protein in the striatum and cortex of animals exposed to treadmill exercise, a finding that coincided with significant increases in blood vessel density in the striatum. Although NGF is most notably important for neurogenesis, the findings that NGF is activated in response to exercise and that its increased expression coincides with significant angiogenesis, suggest that neurogenesis and angiogenesis are overlapping processes and that in addition to VEGF and BDNF, NGF may also be involved in exercise-induced angiogenesis.

Using a slightly different exercise training paradigm, Radak *et al.* [172] exposed rats to prolonged periods of swimming exercise (60–120 min) for eight weeks, and some animals were then “detrained” for another eight weeks during which they did not exercise. They found a trend toward significant increases in hippocampal NGF in the swimming animals compared to sedentary control rats, but this increase was abolished after eight weeks of detraining, when levels of NGF decreased to below control levels. 

### 4.4. Insulin-Like Growth Factor 1 (IGF-1)

IGF-1 is a growth factor that is involved in several processes in during development and in adult organisms [208]. For example, IGF-1 is known to be activated during central nervous system development in such processes as cell proliferation and differentiation [209]. In adult animals, IGF-1 is involved in similar processes that are important for the formation and persistence of new neurons [208]. There is also evidence that IGF-1 is neuroprotective following brain injuries such as stroke and in neurodegenerative diseases [103,210]. Other research has also identified several ways in which IGF-1 is involved in the brain’s response to exercise.

To show that IGF-1 is involved in adult hippocampal neurogenesis, Trejo, Carro, and Torres-Aleman [211] injected normal (non-exercising) rats with both BrdU to mark actively dividing neurons, and an infusion of either an IGF-1 antagonist or saline. They found that animals infused with IGF-1 had significantly greater BrdU-positive neurons compared to animals infused with saline. To further investigate the effects of IGF-1 on hippocampal neurogenesis, and to determine the molecule’s role in exercise-induced neural plasticity, these authors also exercised animals on a treadmill for one hour per day for two weeks, during which they blocked the activity of IGF-1 by way of an infusion of IGF-1 antiserum. Results showed that exercising animals that received the infusion of IGF-1 antiserum had significantly fewer BrdU-labeled cells in the hippocampus compared to exercising animals that had received a control infusion. In other words, blocking IGF-1 activity abrogated any increases in hippocampal neurogenesis as a result of exercise, thus suggesting that IGF-1 is required for normal activity-induced increases in neuron number.

Another study reported similar findings to those of Trejo *et al.* [211] in exercising mice in regard to hippocampal neurogenesis and IGF-1 blockade [212]. Specifically, mice were exposed to two weeks (40 min per day) of treadmill exercise and treated with either an IGF-1 antagonist or with a control infusion. Exercising animals treated with the IGF-1 antagonist had significantly fewer BrdU-positive cells in the hippocampus compared to exercising animals treated with a control infusion. In addition, they found that exercise significantly increased the density of dendritic spines in both the dentate gyrus and in area CA1 (on basal dendrites only), but had no effect on spines in area CA3. However, treatment with the IGF-1 blocking infusion did not abolish the exercise-induced increases in spine density in these areas. These findings suggest that whereas exercise-induced neurogenesis is dependent upon the activity of IGF-1, exercise-induced alterations in spine density in select areas of the hippocampus does not appear to require the activity of IGF-1 [212]. 

The relationship between IGF-1 and other exercise-induced changes in brain anatomy and physiology have also been investigated. Some research has shown that IGF-1 is needed for exercise-induced angiogenesis [173]. Specifically, mutant mice that produced very low levels of IGF-1 failed to undergo significant changes in blood vessel density in response to exercise. Normal animals that exercised, on the other hand, had significantly greater blood vessel density in both the cerebellum and hippocampus compared to normal sedentary animals and mutant exercising animals. These authors also found that if injected with IGF-1, the exercising mutant mice did show significant increases in blood vessel density to a similar degree as normal exercising animals, which further supports the hypothesis that IGF-1 is required for the growth of blood vessels in the brain following exercise [173]. 

Overall, research regarding the morphological changes to the brain and their relationship to the activity of IGF-1 indicate that this growth factor is vital to supporting exercise-induced brain plasticity. Both activity-induced neurogenesis [211,212] and activity-induced angiogenesis [173] have been shown to be negatively affected when the actions of IGF-1 are blocked or reduced. Along with several other growth factors that play prominent roles in exercise-induced brain plasticity, IGF-1 is another growth factor that functions to change the brain in positive ways in physically active organisms.

## 5. Conclusions

Exercise affects both humans and non-human animals in a variety of ways. In addition to improving depression, anxiety and self-esteem in both children and young adults, physically active older adults appear to have increased protection against cognitive decline. While the exact contribution of exercise to mental health and cognitive function is unknown, a number of candidate cellular mechanisms, including neurogenesis, angiogenesis and apoptosis appear to be involved. There are also several molecular mediators of the effects of exercise on the makeup of the brain. Specifically, the expression patterns of growth factors are significantly altered in response to physical activity and exert significant downstream effects on brain physiology and anatomy. Furthermore, these exercise-induced molecular pathways have been implicated in behavioral changes, most notably increased performance on cognitive tasks. The fact that exercise-induced changes in brain anatomy or physiology are accompanied by changes in behavioral performance highlights the importance of continued research regarding the ways in which physical activity can benefit various populations of human and non-human animals. From young organisms to those of advanced age, and from neurologically healthy individuals to those that have suffered any variety of neural insults, exercise has the potential to provide significant improvements in cognitive functioning and furthermore, quality of life. It is imperative that neuroscientists continue to examine the molecular, structural, and anatomical correlates of exercise and behavior.
